# Inequalities in antenatal care in Ghana, 1998–2014

**DOI:** 10.1186/s12884-022-04803-y

**Published:** 2022-06-13

**Authors:** Abdul-Aziz Seidu, Joshua Okyere, Eugene Budu, Henry Ofori Duah, Bright Opoku Ahinkorah

**Affiliations:** 1 Department of Population and Health, University of Cape Coast, Cape Coast, Ghana; 2 College of Public Health, Medical and Veterinary Sciences, James Cook University, Townsville, QLD Australia; 3 Research Department, FOCOS Orthopaedic Hospital, Accra, Ghana; 4 School of Public Health, Faculty of Health, University of Technology, Sydney, Australia

**Keywords:** Antenatal care, Inequality, Ghana, Demographic and Health Surveys, Global health

## Abstract

**Background:**

In order to effectively and efficiently reduce maternal mortality and ensure optimal outcomes of pregnancy, equity is required in availability and provision of antenatal care. Thus, analysis of trends of socio-economic, demographic, cultural and geographical inequities is imperative to provide a holistic explanation for differences in availability, quality and utilization of antenatal care. We, therefore, investigated the trends in inequalities  in four or more antenatal care visits in Ghana, from 1998 to 2014.

**Methods:**

We used the World Health Organization’s (WHO) Health Equity Assessment Toolkit (HEAT) software to analyse data from the 1998 to 2014 Ghana Demographic and Health Surveys. We disaggregated four or more antenatal care visits by four equality stratifiers: economic status, level of education, place of residence, and sub-national region. We measured inequality through summary measures: Difference, Population Attributable Risk (PAR), Ratio, and Population Attributable Fraction (PAF). A 95% uncertainty interval (UI) was constructed for point estimates to measure statistical significance.

**Results:**

The Difference measure of 21.7% (95% UI; 15.2–28.2) and the PAF measure of 12.4% (95% UI 9.6–15.2) indicated significant absolute and relative economic-related disparities in four or more antenatal care visits favouring women in the highest wealth quintile. In the 2014 survey, the Difference measure of 13.1% (95% UI 8.2–19.1) and PAF of 6.5% (95% UI 4.2–8.7) indicate wide disparities in four or more antenatal care visits across education subgroups disfavouring non-educated women. The Difference measure of 9.3% (95% UI 5.8–12.9) and PAF of 5.8% (95% UI 4.7–6.8) suggest considerable relative and absolute urban–rural disparities in four or more antenatal care visits disfavouring rural women. The Difference measure of 20.6% (95% UI 8.8–32.2) and PAF of 7.1% (95% UI 2.9–11.4) in the 2014 survey show significant absolute and relative regional inequality in four or more antenatal care  visits, with significantly higher coverage among regions like Ashanti, compared to the Northern region.

**Conclusions:**

We found a disproportionately lower uptake of four or more antenatal care visits among women who were poor, uneducated and living in rural areas and the Northern region. There is a need for policymakers to design interventions that will enable disadvantaged subpopulations to benefit from four or more antenatal care visits to meet the Sustainable Development Goal  3.1 that aims to reduce the maternal mortality ratio (MMR) to less than  70/100, 000 live births by 2030. Further studies are essential to understand the underlying factors for the  inequalities in antenatal care visits.

## Background

Since the introduction of the Millennium Development Goals (MDGs) in 2000, the world saw a significant decline in maternal mortality ratio (MMR) from a 1.2% annual decrease between 1990 and 2000 to a 3.0% decrease between 2005 and 2015 [1, 2]. Despite these significant improvements in global MMR, low-and middle-income countries (LMICs), also within sub-Saharan Africa (sSA), continue to have high MMRs. Alkema et al. [3] postulated that sSA alone accounted for 66% of the global burden of maternal deaths in 2015. In Ghana, maternal mortality is a public health issue with an estimated MMR of 310 per 100,000 live births in 2017 [4].

One of the interventions that have been identified to be effective in reducing maternal mortality, morbidity and disability is antenatal care [ANC] [5]. Hence, WHO recommends a minimum of four ANC visits (from 2016 onwards increased to 8 visits). One purpose of ANC attendance is for early detection of potential complications, which allows health care providers to initiate interventions to mitigate the associated risks to reduce the risk of maternal mortality and severe morbidity [6]. This implies that failure to attend ANC at least 4 times may result in complications or even worsen already existing complications and potentially lead to the death of the mother or child or both.

It must be noted, however, that ANC attendance does not occur in isolation; it is dependent on a myriad of predisposing factors such as geographical, socioeconomic, religious, cultural, and demographic factors [7, 8]. These factors are in themselves inequalities that restrict pregnant women’s capacity to go through the required minimum of four ANC visits. In the case of Ghana, factors such as parity, religion, wealth, age, residence, cost of ANC as well as the quality and timing of ANC have been recognised to be influential on pregnant women’s ability to access four or more ANC visits [9, 10].

In order to effectively and efficiently avert or reduce the risk of adverse maternal health outcomes ( i.e., morbidity, disability and mortality), equality in the availability and provision of ANC is required. Evidence from the 2016 Lancet Global Burden of Disease study suggests that “achievement of the Sustainable Development Goal (SDG 3.1) will require 91% coverage of one ANC visit, 78% of four ANC visits, 81% of in-facility delivery, and 87% of skilled birth attendance.” [11]. These percentages may be instrumental to reduce MMRs to 70 per 100,000 live births by 2030. Thus, trend analysis of socio-economic, demographic, cultural and geographical inequities is imperative for a holistic explanation of differences in the availability, quality, and utilisation of ANC. On this premise, we sought to investigate the trends in inequalities in 4 + ANC visits among pregnant women in Ghana, from 1998 to 2014. Our findings may contribute to the knowledge on trends and inequalities in ANC attendance. Again, it would provide a framework to guide policy makers and programme developers to know what aspects of ANC ought to be altered to increase ANC visits and contribute to the reduction of maternal mortality considering the SDG 3 which seeks to reduce the global MMR to less than 70 per 100,000 live births and a neonatal mortality rate below 20 per 1.000 livebirths by 2030 [12].

## Materials and methods

### Study setting

The study setting is the Republic of Ghana, one of the countries in West Africa with a total land area of 238,533 square kilometres [13]. It is bounded by Burkina Faso in the north, Togo in the east and Côte d'Ivoire in the west. According to the different National Population Census (PHC), the population stood at 6,726,815 (1960); 8,559,313 (1970); 12,296,081 (1984); 18,912,079 (2000) and 24,658,823 (2010) [13]. At the time of the surveys, the country was divided into ten regions, which have currently increased to 16.

### Data source

Secondary data obtained from the 1998, 2003, 2008, 2014 Ghana Demographic and Health Survey (GDHS) were used in this study. DHSs are conducted under the MEASURE DHS program in LMICs, mostly every five years since its start. The surveys focus on gathering information on women’s, children’s, men’s and households’ health. For women, some issues are about utilization of maternal health care, such as ANC visits. The survey used a two-stage sampling design, which begins with the selection of clusters across urban and rural locations from the entire nation. These clusters represent enumeration areas demarcated during the Ghana PHC. Next stage is the selection of households from predefined clusters. Details of the methodologies employed in the various rounds can be found in the final reports of GDHS [14]. In this study, only women with live births were considered. The sample sizes for each round of survey were as follows (n = 4141 in 2014, 2098 in 2008, 2643 in 2003 and 2310 in 1998). DHS data are available through the World Health Organization’s (WHO) Health Equity Assessment Toolkit (HEAT) and have two different ANC-visit measures (previous 2/3 years and previous 5 years). We only used the 5-years variable.

### Selection and measures of variables

The outcome variable in this study was the utilization of ANC visits. Women were asked about the number of ANC visits they made during their recent pregnancy. This variable was dichotomized as 0–3 or 4 + ANC visits. Inequality in ANC visits was examined using four equity stratifiers: economic status (wealth index), level of education, place of residence and subnational region. In DHS, wealth index is computed using Principal Component Analysis (PCA) [15]. It is classified as poorest, poorer, middle, richer and richest quintiles. Level of education of the mother was categorized as no education, primary and secondary/higher education. Place of residence was classified as urban vs. rural and subnational regions were grouped into the then ten regions.

### Statistical analyses

Using the 2019 updated WHO’s HEAT version 3.1 software [16], inequality in 4 + ANC visits was carried out in two steps. First, ANC visits were disaggregated by the four equity stratifiers: economic status, level of education, place of residence, and subnational region. This was done to present results on the distribution of the estimates and uncertainty intervals of 4 + ANC visits across the various equity stratifiers. Secondly, inequality was calculated using four measures of inequality: Difference, Population Attributable risk (PAR), Population Attributable Fraction (PAF) and Ratio. Difference and Ratio are simple measures whiles PAR and PAF are complex measures. While Ratio and PAF are relative measures, the remaining two are absolute summary measures. The choice of summary measures was based on evidence suggesting the scientific significance of adopting both absolute and relative summary measures in a single health inequality study [17, 18]. Whereas complex measures account for size of categories of sub-populations, simple measures do not. Notwithstanding, simple measures are easy for interpretation and understanding [17, 18]. Therefore, combining both relative and absolute measures helps to provide a more comprehensive analysis. Detailed procedure and calculation of the summary measures are available in the HEAT software technical notes [16, 17].

## Results

### Trends in prevalence of 4 + ANC visits across economic status, level of education, place of residence and Ghanaian sub-national regions

The prevalence of 4 + ANC visits changed over the years: it was 63.7% in 1998, it increased to 69.3% in 2003, 78.1% in 2008 and 87.2% in 2014. The extent of 4 + ANC visits varied across economic status, with higher concentrations among those in the richest wealth quintile (87.6% in 1998, 91.0% in 2003, 93.8% in 2008 and 98.1% in 2014). In terms of level of education, the prevalence of 4 + ANC visits was higher among women with secondary/higher education in 1998 [77.0%, 95% UI = 73.98–79.80], 2003 [80.7%, 95% UI = 77.69–83.4], 2008 [88.2%, 95% UI = 85.37–90.53] and 2014 [92.9%, 95% UI = 91.41–94.14]. The same trend of higher 4 + ANC visits was observed among women living in urban areas, compared to those in rural areas from 1998–2014. Except in 2014, where the concentration of 4 + ANC visits was higher among women who lived in the Ashanti region [93.5%, 95% UI = 89.54–96.01], the prevalence of 4 + ANC visits showed an increasing trend among women in the Greater Accra region (Table 1). However, the regions with relatively low prevalence of 4 + ANC visits in 2014 were the Volta region [77.3%, 95% UI = 69.2, 83.7], Eastern region [77.4%, 95% UI = 71.8, 82.1] and Northern region [72.3%, 95% UI = 60.3, 82.7]. Overall, we found that the prevalence of 4 + ANC visits in Ghana increased by 23.5% from 63.7% in 1998 to 87.3% in 2014.

**Table 1:** Trends in prevalence of 4 + ANC visits among pregnant women, disaggregated across four inequality dimensions, 1998–2014

Inequality dimension	**1998 (63.7%)**		**2003 (69.3%)**		**2008 (78.1%)**		**2014 (87.2%)**	
	n	Estimate [95% UI]	n	Estimate [95% UI]	n	Estimate [95% UI]	n	Estimate [95% UI]
**Economic status**								
Quintile 1 (poorest)	591	45.7% [41.16, 50.29]	648	55.6 [50.28, 60.74]	479	62.9 [57.61, 67.83]	869	76.40 [69.51, 82.14]
Quintile 2	493	60.5 [55.73, 65.10]	556	62.6 [57.76, 67.16]	461	73.2 [68.34, 77.64]	839	82.1 [78.99, 84.78]
Quintile 3	462	63.2 [58.27, 67.84]	533	66.2 [61.53, 70.50]	400	77.6 [72.75, 81.83]	827	86.8 [83.59, 89.48]
Quintile 4	417	73.9 [68.81, 78.43]	473	80.0 [74.38, 84.71]	436	89.1 [84.00, 92.70]	814	94.1 [91.67, 95.89]
Quintile 5 (richest)	344	87.6 [83.94, 90.52]	432	90.9 [87.59, 93.51]	321	93.8 [89.61, 96.37]	791	98.1 [95.75, 99.13]
**Level of education**								
No education	844	50.7 [47.08, 54.39]	1024	58.5 [54.62, 62.33]	646	68.4 [63.89, 72.59]	1078	79.2 [73.46, 84.02]
Primary school	468	58.8 [53.60, 63.76]	588	68.3 [63.40, 72.78]	511	72.0 [67.53, 76.07]	812	82.3 [78.72, 85.33]
Secondary/higher	997	77.0 [73.98, 79.80]	1031	80.7 [77.69, 83.44]	941	88.2 [85.37, 90.53]	2251	92.9 [91.41, 94.14]
**Place of residence**								
Rural	1708	58.5 [55.43, 61.48]	1698	61.0 [57.96, 63.99]	1255	71.5 [68.03, 74.72]	2228	82.9 [79.52, 85.89]
Urban	602	78.6 [74.62, 82.01]	945	84.3 [81.25, 86.99]	843	88.1 [84.72, 90.74]	1913	92.3 [90.43, 93.78]
**Sub-national Regions **								
Western region	289	64.4 [58.09, 70.29]	246	61.5 [52.95, 69.38]	189	78.0 [69.49, 84.62]	427	92.1 [87.35, 95.16]
Central region	258	66.9 [61.38, 72.13]	211	67.5 [58.22, 75.55]	199	76.6 [66.68, 83.89]	455	91.1 [87.53, 93.71]
Greater Accra region	254	74.7 [65.66, 82.03]	302	80.7 [73.87, 86.22]	262	89.5 [82.84, 93.81]	674	91.4 [87.85, 94.02]
Volta region	257	54.4 [47.59, 61.06]	220	65.3 [56.55, 73.16]	181	74.5 [64.94, 82.15]	315	77.3 [69.20, 83.74]
Eastern region	316	59.7 [50.98, 67.86]	265	63.9 [56.10, 71.13]	185	73.2 [65.81, 79.41]	388	77.4 [71.84, 82.08]
Ashanti region	372	66.4 [59.58, 72.67]	506	79.2 [74.67, 83.01]	395	84.3 [78.75, 88.63]	738	93.5 [89.54, 96.01]
Brong Ahafo region	189	68.4 [60.06, 75.73]	296	75.9 [69.91, 81.08]	217	80.2 [69.57, 87.81]	374	90.3 [85.36, 93.68]
Northern region	150	42.0 [32.55, 52.09]	346	54.1 [47.55, 60.53]	291	68.3 [61.65, 74.26]	479	72.9 [60.33, 82.72]
Upper West region	68	56.7 [46.77, 66.05]	83	64.5 [54.71, 73.20]	118	66.9 [55.31, 76.74]	177	93.0 [89.91, 95.20]
Upper East region	153	74.6 [67.63, 80.50]	165	69.1 [56.41, 79.47]	57	83.6 [77.71, 88.20]	110	91.3 [86.00, 94.74]

### Trends of 4 + ANC visits indicating the magnitude of inequality

Table 2 shows the socio-economic, urban–rural, educational and subpopulations disparities in 4 + ANC visits from 1998 and 2014, favouring women who are socio-economically advantaged, those with higher educational attainment, urban residents and those living in regions like the Greater Accra. More precisely, we found substantial absolute (D, PAR) and relative (R, PAF) wealth-driven disparities in 4 + ANC visits over the 16 years with higher coverage among advantaged subpopulations such as richest and richer women as compared to poorest and poorer. For instance, in the 2014 survey, the Difference measure of 21.7% [95% UI; 15.12–28.15] and the PAF of 12.4% [95% UI; 9.62–15.16] indicate significant absolute and relative economic-related disparities in 4 + ANC visits favouring economically advantaged women. Significant absolute and relative education-related disparities in 4 + ANC visits were discovered from 1998 to 2014 using all four summary measures, disfavouring non-educated subpopulations. For example, in the 2014 survey, the Ratio and PAR measures of 1.2% [95% UI; 1.09–1.25] and 5.6% [95% UI; 3.66–7.63] both indicate wide disparities in 4 + ANC visits across education subgroups disfavouring non-educated women.

**Table 2:** Inequality indices estimates of the factors associated with prevalence 4 + ANC visits among pregnant women, 1998–2014

Inequality Dimension	1998			2003			2008			2014		
	Estimate	LB	UB	Estimate	LB	UB	Estimate	LB	UB	Estimate	LB	UB
**Economic status**												
Difference	41.91	36.32	47.50	35.41	29.43	41.38	30.94	24.88	37.00	21.66	15.17	28.15
PAF	37.48	32.00	42.96	31.17	26.41	35.93	20.03	15.27	24.79	12.39	9.62	15.16
PAR	23.88	20.39	27.37	21.62	18.32	24.92	15.65	11.93	19.38	10.81	8.39	13.22
Ratio	1.92	1.71	2.12	1.64	1.47	1.80	1.49	1.36	1.62	1.28	1.18	1.39
**Level of education**												
Difference	26.28	21.63	30.93	22.20	17.41	26.98	19.79	14.75	24.84	13.67	8.23	19.10
PAF	20.88	16.67	25.09	16.40	13.06	19.73	12.85	9.16	16.54	6.47	4.19	8.74
PAR	13.30	10.62	15.99	11.37	9.06	13.69	10.04	7.16	12.92	5.64	3.66	7.63
Ratio	1.52	1.40	1.64	1.38	1.28	1.48	1.29	1.20	1.38	1.17	1.09	1.25
**Place of residence**												
Difference	20.05	15.30	24.81	23.32	19.18	27.45	16.56	12.09	21.04	9.33	5.76	12.91
PAF	23.27	21.49	25.05	21.60	19.72	23.47	12.67	10.85	14.50	5.76	4.68	6.83
PAR	14.83	13.69	15.96	14.98	13.68	16.28	9.90	8.48	11.33	5.02	4.09	5.96
Ratio	1.34	1.25	1.44	1.38	1.30	1.46	1.23	1.16	1.30	1.11	1.07	1.16
**Sub-national Region **												
Difference	32.71	19.92	45.50	26.68	17.76	35.61	22.64	10.56	34.71	20.52	8.82	32.23
PAF	17.26	5.18	29.33	16.49	9.45	23.54	14.57	4.06	25.07	7.14	2.94	11.35
PAR	10.99	3.30	18.69	11.44	6.55	16.33	11.38	3.18	19.59	6.23	2.56	9.90
Ratio	1.78	1.32	2.24	1.49	1.28	1.70	1.34	1.11	1.57	1.28	1.08	1.48

Difference, *PAR* Population Attributable risk, *PAF* Population Attributable Fraction and Ratio. Difference and Ratio are relative measures whiles PAR and PAF are absolute summary measures

Furthermore, this study shows extensive anti-rural inequalities in 4 + ANC visits using both absolute (Difference, PAR) and relative (Ratio, PAF) measures over the 16 year period. For example, the PAF measure of 5.8% [95% UI; 4.68–6.83] and PAR measure of 5.0% [95% UI; 4.09- 5.96] suggest considerable relative and absolute urban–rural disparities in 4 + ANC visits disfavouring rural women. The results further show sizeable subnational inequality in 4 + ANC visits over the 16 years using absolute (Difference, PAR) and relative (Ratio, PAF) measures. The Difference measure of 20.6% [95% UI; 8.82–32.23] and PAF of measure 7.1% [95% UI; 2.94–11.35] in the 2014 survey, show significant absolute and relative regional inequality in 4 + ANC visits, with significantly higher coverage among regions like Ashanti.

## Discussion

This study shows an increasing trend in the prevalence of 4 + ANC visits from 63.7% in 1998 to 87.2% in 2014 in Ghana. This increasing trend could reflect state policies and interventions such as the free maternal healthcare policy and national health insurance scheme which are pro-maternal healthcare [19]. Another plausible explanation for this observation could be a resultant effect of the increased presence of Community-Based Health Planning and Services (CHPS) across the country since 1999. Also, we found the prevalence of 4 + ANC visits to be relatively higher among women who were economically advantaged, those with at least secondary education, urban residents, and those living in the Greater Accra and Ashanti regions.

In 2014, the prevalence of 87.3% is higher than the target of 78% recommended in the 2016 Lancet Global Burden of Diseases Study for the attainment of SDG 3, but certain socio-economic disparities could undo the gains made over the 16-years. The 2014 prevalence was higher than the 51% reported in Nigeria, 58.2% in Benin Republic, 78.1% in Liberia, 76% in Sierra Leone, 70.4% in Lesotho, and 64.8% in Zimbabwe [20, 21]. A key contributing factor to the 23.5% increase in the prevalence over the 16-year period could be the introduction of the free maternal health policy by the government of Ghana in 2008 to bridge financial access and inequality gaps [19].

In support of previous findings in Ghana, Malawi, Gabon, Nigeria, sSA and Pakistan, we found that women in the richest wealth quintile had higher prevalence of at least 4 ANC visits during the 16-year period [6, 22–27]. Despite the free ANC services in Ghana, there are other associated costs, such as transport to health facilities and medications, that are not covered by the national health insurance and could serve as potential barriers for women with low economic status [6]. However, the finding was incongruent with findings from a community-based survey in North Eastern Ethiopia where no economic inequalities were found with regard to 4 + ANC visits [28]. These divergences could be a result of socio-demographic differences in the two study populations.

Women with at least secondary education were consistently more advantageous in accessing 4 + ANC  compared to those with less than secondary education. This aligns with the findings of previous studies in Ghana that revealed that education significantly influences ANC visits [5, 29–31]. Since education is known to increase health literacy and utilization, it may explain the reason for this observation [32, 33]. Additionally, educated women are more financially independent and may have better income to access ANC [34].

Women residing in urban areas had a higher prevalence of at least four ANC visits compared to those in rural areas. Previous studies have also reported significant positive associations between place of residence and ANC visits [35–40]. Even though Ghana has a free ANC program, there are reports of inequalities in the distribution of both skilled health workers and health facilities with respect to rural–urban areas [30]. Women in urban areas are more advantageous with respect to the availability of advanced health facilities and skilled health attendants. Conversely, women in rural areas have inadequate, unequipped, and often distant health facilities with few skilled health attendants leading to ANC drop-outs and high patronage of traditional birth attendants [30]. The Greater Accra and Ashanti Regions are predominantly urban with the two most densely populated cities in Ghana whereas the Northern Region is predominantly rural and one of the poorest regions in Ghana [12]. Therefore, the findings of our study imply the need for improvement in 4 + ANC visits not only in the Northern region but also in the Eastern and Volta regions where ANC visits were lower than the Lancet Report’s 78% attendance goal [11].

### Strengths and limitations

The strength of this paper lies in its methodology. The study used nationally representative GDHS which allows generalization to all Ghanaian women. The use of both absolute and relative inequality measures provides multi-dimensionality to viewing the magnitude and trends of inequality in ANC visits. Also, this study presented the results through four equity stratifiers: economic status, level of education, place of residence, and sub-national region. As such, government, policy makers as well as responsible agencies can easily identify priority areas in revamping policies and interventions geared towards an increase in the proportion of 4 + ANC visits. Again, this will enable interventionists to optimize their resources in promoting 4 + ANC visits because they can now identify specific subpopulations and how to focus interventions towards the achievement of SDG 3.1 that seeks to reduce global MMR to less than 70 per 100,000 live births by 2030. Despite these strengths, there are some inherent limitations which should be considered. Our dataset was based on cross-sectional design; hence causal inferences cannot be established. Also, the results are entirely descriptive and ecological, and therefore no causal association can be inferred.

## Conclusion

Evidence on inequities in 4 + ANC visits in Ghana was observed and a disproportionately lower uptake was found among women who were poor, uneducated, living in rural areas and the Northern region. There is a need for policy makers to design interventions that will enable disadvantaged subpopulations to benefit from ANC with 4 + visits to meet SDG 3.1 and 3.2, which aim to reduce MMR to less than 70/100, 000 live births and a neonatal mortality rate to as low as 12 per 1000 live births by 2030, respectively. Further studies are essential to understand the underlying factors for the observed inequalities in ANC attendance.

## Data Availability

The datasets generated and/or analyzed during the current study are available in the WHO’s HEAT version 3.1 [https://www.who.int/gho/health_equity/assessment_toolkit/en/].

## Abbreviations

ANC: Antenatal care
EA: Enumeration Area
GDHS: Ghana Demographic and health Survey
HEAT: Health Equity Assessment Toolkit
LMICs: Low- and middle-income countries
MMR: Maternal mortality ratio
PAF: Population Attributable Fraction
PAR: Population Attributable Risk
PCA: Principal Component Analysis
PPS: Probability Proportional to Size
SDG: Sustainable Development Goal
UI: Uncertainty Intervals
WHO: World Health Organization
